# The effect of coronavirus disease 2019 vaccination on pregnant women: A scoping review

**DOI:** 10.4102/hsag.v29i0.2577

**Published:** 2024-12-06

**Authors:** Mildred M. Malamule, Rodwell Gundo, Mavis Mulaudzi

**Affiliations:** 1 Department of Nursing Science, Faculty of Health Science, University of Pretoria, Pretoria, South Africa

**Keywords:** COVID-19, vaccination, pregnant women, pregnancy, SARS-CoV-2

## Abstract

**Background:**

Globally, reports have shown that pregnant women refuse to receive the coronavirus disease 2019 (COVID-19) vaccine. This has posed a significant concern given the global impact of the COVID-19 pandemic.

**Aim:**

This study aims to explore the current evidence on the effect of COVID-19 vaccination on pregnant women.

**Method:**

A scoping review was conducted using Levac et al.’s five-stage framework. Relevant articles were searched in the Web of Science, PubMed, Scopus and EBSCOhost (CINAHL) databases. The identified articles were screened based on predetermined inclusion and exclusion criteria. Data from the selected articles were charted and summarised into meaningful units.

**Results:**

Twelve articles from developed countries were included in the review. Studies have reported that COVID-19 vaccination during pregnancy is generally safe and does not increase the risk of pregnancy complications. There was no significant difference in delivery outcomes between vaccinated and unvaccinated women. Neonatal outcomes were not affected by the vaccination. However, one study identified a potential risk of spontaneous abortion between 6 and 9 weeks of gestation among vaccinated women.

**Conclusion:**

Coronavirus disease 2019 vaccination is considered safe during pregnancy. While some studies have identified potential associations with certain conditions, the overall benefits of vaccination outweigh the risks. Continued monitoring of the safety and effectiveness of COVID-19 vaccines during pregnancy is recommended. Pregnant women should consult healthcare providers to make informed decisions regarding vaccination.

**Contribution:**

The findings of this review may assist in alleviating anxiety and reducing vaccine hesitancy among pregnant women.

## Introduction

Coronavirus disease 2019 (COVID-19) has become a major public health challenge worldwide since it was declared a pandemic in 2020. Cucinotta and Vanelli (2020) defined COVID-19 as a respiratory infection caused by severe acute respiratory syndrome coronavirus 2 virus (SARS-CoV-2). The first case of COVID-19 was detected in Wuhan, China, in December 2019, after which the virus spread worldwide (Hussain et al. 2021). As of early 2022, the World Health Organization reported 489 million cases and over 6 million deaths globally (WHO 2022). Despite the implementation of preventative measures, the number of new cases and deaths increased daily. Therefore, the vaccines represented a significant breakthrough because no antiviral treatment has been scientifically proven to treat infection (Egloff et al. 2022).

Although pregnant women were not included in the initial vaccine trial, the American College of Obstetricians and Gynaecologists and other organisations recommended that pregnant women receive the vaccine as it is considered safe during pregnancy (Halasa et al. 2022). Studies have indicated that receiving the vaccine during pregnancy reduces the risk of maternal complications such as preterm birth and stillbirth (Carbone et al. 2022). Furthermore, the vaccine protects infants under 6 months from COVID-19-related hospitalisation (Halasa et al. 2022). In addition, it protects infants from COVID-19 infection in their first 4 months of life when they are not yet mature enough to receive the vaccine (Carlsen et al. 2022).

Despite governments’ efforts to promote vaccination among pregnant women, vaccine hesitancy has been reported as a significant concern worldwide. Studies have reported that pregnant women are more reluctant to take the vaccine because of the fear of side effects and safety issues (Kalra et al. 2023). Pregnant women who remain unvaccinated and contract COVID-19 are at high risk of hospitalisation in intensive care units (ICUs). This finding aligns with the findings of Engjom et al. (2022), which reported a higher likelihood of caesarean section because of foetal distress or other pre-existing conditions among unvaccinated women. In addition, infants born to unvaccinated mothers may be at high risk of COVID-19 respiratory complications, which may require ICU hospitalisation (Halasa et al. 2022).

There is a need to explore the available evidence on the safety, effects on pregnancy outcomes and neonatal outcomes of COVID-19 vaccination in pregnant women. These findings may contribute to the body of knowledge on COVID-19 vaccination and ultimately inform decisions by pregnant women and healthcare professionals. Globally, numerous studies have investigated the safety of COVID-19 vaccine among pregnant women. However, no scoping review has investigated the effects of COVID-19 vaccination on pregnant women. Therefore, this review was conducted to explore and describe the impact of the COVID-19 vaccine on pregnant women. A preliminary search for existing scoping reviews on the effects of COVID-19 vaccination on pregnant women was conducted and no reviews were identified.

## Methods

### Research design

A scoping review aimed at summarising the effect of COVID-19 vaccination on pregnant women was conducted following the five stages proposed by Levac, Colquhoun and O’Brien (2010). The steps include identifying the research question, identifying relevant studies, selecting studies, charting the data, collating, summarising and reporting the results. This review was conducted to map the available evidence on the effects of COVID-19 vaccination on pregnant women. The presentation of the results was guided by the Preferred Reporting Items for Systematic Reviews and Meta-Analyses Extension for Scoping Reviews (PRISMA-ScR) (Tricco et al. 2018). This scoping review was not registered on any platform.

### Identifying the research question

This study used the population, concept, and context (PCC) framework (see Table 3) as recommended by the Joanna Briggs Institute (JBI) to identify the main concepts in the review question and to inform the research strategy (Pollock et al. 2023). Therefore, the research question was as follows: ‘*What is currently known about the effect of the COVID-19 vaccine on pregnant women?*’

### Identifying relevant studies

In consultation with a specialist librarian, the authors searched the following databases: Web of Science, PubMed, Scopus and EBSCOhost (CINAHL). A search strategy was developed using the same concepts in both databases. Concepts or terms that were used in different combinations, as per Boolean phrases, were as follows: ‘Impact’ OR ‘Effect’ OR ‘Influence’ OR ‘Consequences’ OR ‘Benefits’ AND ‘COVID-19 Vaccine’ OR ’Coronavirus vaccine’ OR ‘Coronavirus vaccine’ OR ‘SARS vaccine’ OR ‘SARS 2 vaccine’ OR ‘COVID-19 vaccines’ OR ‘COVID-19 vaccination’ AND ‘Pregnant Women’ OR ‘Expecting mother’ OR ’Pregnancy’. The search results were then exported to Rayyan for screening (Harrison et al. 2020). The last search date for this review was 15 May 2023. The entire search strategy has been added as supplementary material.

### Study selection

**FIGURE 1:**
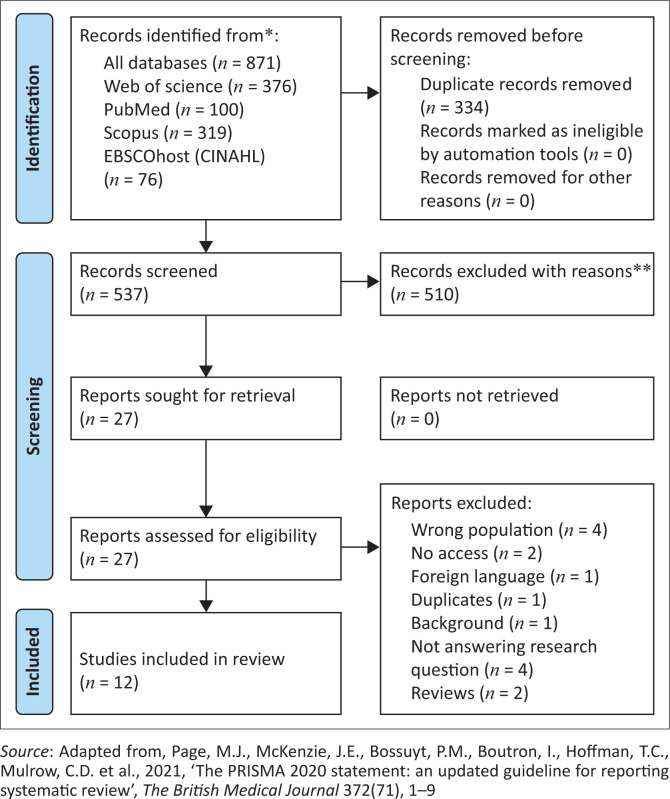
Preferred Reporting Items for Systematic Reviews and Meta-Analyses diagram of the search process and inclusion criteria.

After the search process was completed, two authors independently screened the titles and abstracts on the basis of the inclusion and exclusion criteria. The relevant studies were then subjected to full-text screening by the two authors. When there was disagreement between the two authors, the third author was invited to discuss and resolve the differences. The PRISMA flow diagram in Figure 1 illustrates the process.

#### Inclusion criteria

The review included articles published in English between 2020 and 2023 because the first case of COVID-19 was reported in December 2019. Studies were included if pregnant women comprised the study population – vaccinated and unvaccinated against COVID-19 – and if they examined the effect of the COVID-19 vaccine on pregnant women or pregnancy outcomes following vaccination. Studies conducted in both developed and undeveloped countries were included.

#### Exclusion criteria

The following articles were excluded from the review: articles that focused on the general population rather than on pregnant women, articles that did not answer the review question, articles that did not mention pregnant women or the COVID-19 vaccine and articles that were published in languages other than English. In addition, articles published without full text, review articles, commentaries and editorial letters were excluded.

### Charting the data

Two authors independently extracted the relevant data from the 12 included articles via a Microsoft Excel data extraction spreadsheet. The following information was extracted from each article: the author’s name, year of publication, country of origin, study aim, population and sample size, methodology and key findings. The PCC framework guided data extraction.

### Collating, summarising and reporting the results

This review focused on the effect of COVID-19 vaccination on pregnant women; therefore, this step involved descriptive mapping of the results related to safety, pregnancy outcomes and neonatal outcomes. A summary of the results is presented in Table 1 and Table 2.

**TABLE 1:** Characteristics of the included studies.

Author and publication year	Title	Aim	Study population and sample size	Country	Study design
Komine-Aizawa et al. (2022)	The vaccination status and adverse effect of COVID-19 vaccine among pregnant women in Japan in 2021	To investigate the vaccination status and adverse event reaction to the COVID-19 vaccine among pregnant women in Japan	A total of 6 576 pregnant women: vaccinated (5 397), unvaccinated (1 179)	Japan	Cross-sectional study
Rottenstreich et al. (2022)	The COVID-19 vaccination during the third trimester of pregnancy: immunisation rate and maternal and neonatal outcome, a multicentre retrospective cohort study	To evaluate the impact of COVID-19 vaccination rate (Pfizer-BioNTech BNT162b2) during the third trimester of pregnancy on maternal and neonatal outcomes	A total of 1 775 women 18 years and older, both vaccinated (712) and unvaccinated (1 063), who delivered between 19 January and 27 April 2021, who never tested positive for COVID-19	Jerusalem	Retrospective cohort database study
Mansour et al. (2023)	mRNA COVID-19 vaccination early in pregnancy and the risk of spontaneous abortion in an international pregnancy registry	To evaluate the effect of BNT162b2 vaccination during the first 20 weeks of pregnancy	A total of 6 840 pregnant women who received at least one dose of mRNA COVID-19 vaccine before 20 weeks gestation between January and September 2021	United States	Prospective cohort study
Arulappen et al. (2022)	A multicentre cohort study on the adverse effect evaluation after messenger RNA COVID-19 vaccination among pregnant women healthcare employees in Penang general hospitals	To establish the safety data in real-time through rigorous, proactive data collection to record vaccine-related symptoms and obstetric outcomes.	A total of 121 pregnant employees (healthcare employees) who gave consent for mRNA vaccine	Penang, Malaysia	Retrospective cohort study
Kugelman et al. (2023)	Safety of COVID-19 vaccination in pregnant women: a study of the adverse perinatal outcomes	To examine whether the risk of adverse perinatal outcomes was equivalent among vaccinated and unvaccinated pregnant women	A total of 1 894 women with singleton pregnancy and over 23 weeks and those who were admitted for delivery. Vaccinated (930) Unvaccinated (964)	Israel	Retrospective equivalence cohort
Bleicher et al. (2021)	Early exploration of COVID-19 vaccination safety and effectiveness during pregnancy: interim descriptive data from a prospective observation	To compare short-term outcomes in vaccinated and non-vaccinated pregnant women	A total of 326 pregnant women Vaccinated (202) Unvaccinated (124)	Finland	Prospective observational cohort study
Almutairi et al. (2023)	The COVID-19 vaccine during pregnancy and perinatal outcome	To investigate the effect of COVID-19 vaccine on pregnant women and perinatal outcome	A total of 365 pregnant women: Vaccinated (289) Unvaccinated (76)	Saudi Arabia	Cross-sectional study
Condon et al. (2023)	The impact of COVID-19 vaccination on stillbirth rates among pregnant women in the Metro-Detroit area	To examine the impact of COVID-19 vaccination on intrauterine foetal death at a large multihospital health care system in metropolitan Detroit	A total of 13 368 pregnant women: Vaccinated (12 767), Unvaccinated (502)	Metropolitan Detroit	Retrospective cohort study
Fell et al. (2022)	Association of COVID-19 vaccination in pregnancy with adverse peripartum outcomes	To evaluate peripartum outcomes following COVID-19 vaccination during pregnancy	Better Outcomes Registry and Network Ontario birth registry14 linked with provincial COVID-19 immunisation database (COVaxON) of 97 590: Vaccinated (67 475) and Unvaccinated (30 115)	Ontario, Canada	Retrospective cohort study
Dick et al. (2022)	Safety of SARS-CoV-2 vaccination during pregnancy-obstetric outcomes from a large cohort study	To examine the association between SARS-CoV-2 vaccination during pregnancy and maternal and neonatal outcomes in a large cohort study.	A total of 5 618 Pregnant women who delivered between December 2020 and July 2021. Vaccinated (2 395) Unvaccinated (3 313)	Jerusalem	Retrospective cohort study
Wainstock et al. (2021)	Prenatal maternal COVID-19 vaccination and pregnancy outcomes	To study the association between prenatal Pfizer-BioNTech COVID-19 vaccination, pregnancy course and outcomes	A total of 4 399 Pregnant women who delivered from January to June 2021. Vaccinated (913) Unvaccinated (3 486)	Israel	Retrospective cohort study
Morgan et al. (2023)	Pregnancy outcomes in patients after completion of the mRNA coronavirus disease 2019 (COVID-19) vaccination series compared to unvaccinated patients	To compare the frequency of perinatal death between patients who completed the mRNA vaccination series and unvaccinated patients.	A total of 15 867 Pregnant patients who delivered after 20 weeks of gestation between January and December 2021. Vaccinated (2 069) Unvaccinated (13 796)	United States	Retrospective cohort study

*Source*: Adapted from, Pollock, D., Peters, M.D., Khalil, H., Mcinerney, P., Alexander, L., Tricco, A.C. et al., 2023, ‘Recommendations for the extraction, analysis, and presentation of results in scoping reviews’, *JBI Evidence Synthesis* 21(3), 520–532. https://doi.org/10.11124/JBIES-22-00123

Note: Please see the full reference list of this article for details on the articles cited: https://doi.org/10.4102/hsag.v29i0.2577 for more information.

COVID-19, coronavirus disease 2019; SARS-CoV-2, severe acute respiratory syndrome coronavirus 2.

**TABLE 2:** Pregnancy, delivery and neonatal outcomes.

Author	Pregnancy outcomes	Delivery outcomes	Neonatal outcomes
Dick et al. (2022)	Hypertensive disorder in pregnancy: vaccinated 25 (1.1%), unvaccinated 44 (1.3%) Gestational diabetes: vaccinated 222 (9.6%), unvaccinated 275 (8.3%) Intrauterine foetal death: vaccinated 20 (0.87%), unvaccinated 33 (1.0%)	Postpartum haemorrhage: vaccinated 79 (3.4%), unvaccinated 104 (3.1%) Caesarean section: vaccinated 358 (15.5%) vaccinated, unvaccinated 529 (16.0%)	Small for gestational age: vaccinated 142 (6.2%), unvaccinated 233 (7.0%) 5 min Apgar score < 7: vaccinated 42 (1.8%), unvaccinated 63 (1.9) Preterm birth: vaccinated 127 (5.5), unvaccinated 204 (6.2%)
Rottenstreich et al. (2021)	Chorioamnionitis: vaccinated 2%, unvaccinated 2.4% Placenta abruption: vaccinated 1.1%, unvaccinated 2.4%	Postpartum haemorrhage: vaccinated 7.3%, unvaccinated 10% Caesarean section: vaccinated 15.6% vaccinated, unvaccinated 10.8% Meconium-stained amniotic fluid: vaccinated 14.5, unvaccinated 16.2%	NICU admission: vaccinated 4.1%, unvaccinated 4.5% 5 min Apgar score < 7: vaccinated 2.9%, unvaccinated 2.5% Birth asphyxia: vaccinated 0.2%, unvaccinated 0.9% Hypoglycaemia: vaccinated 2.4%, unvaccinated 2.45
Arulappen et al. (2022)	Intrauterine growth restriction: vaccinated 0%	Caesarean section 37 (31.4%) Vaginal delivery 83 (68.3%)	Preterm birth 14 (11.7%) NICU admission 27 (11.7) Neonatal death 0 (0%)
Bleicher et al. (2021)	Antepartum haemorrhage: vaccinated 5.6%, unvaccinated 1.9% Hypertensive disorder: vaccinated 0%, unvaccinated 0% Pregnancy loss: 14–28 weeks: vaccinated 0%, unvaccinated 0%	-	Preterm birth: vaccinated 0%, unvaccinated 0%
Komine-Aizawa et al. (2022)	Gestational hypertension: vaccinated 0.7%, unvaccinated 0.9% Anaemia: vaccinated 12.5%, unvaccinated 11.1% Gestational diabetes mellitus: vaccinated 4.2%, unvaccinated 3.2%	-	-
Kugelman et al. (2023)	Gestational diabetes mellitus: vaccinated 6.2%, unvaccinated 6.2%	Vaginal delivery: vaccinated 72.2%, unvaccinated 72.5%	-
Morgan et al. (2023)	Preeclampsia: vaccinated 8.1%, unvaccinated 8.2%	-	Neonatal death: vaccinated 0.5%, unvaccinated 0.7%. NICU admission: vaccinated 11.8%, unvaccinated 15.3%. Preterm delivery: vaccinated 10.3%, unvaccinated 13.2%. Very low birth weight: vaccinated 1.1%, unvaccinated 2.1% Small for gestational age: vaccinated 4.1%, unvaccinated 4.7%
Wainstock et al. (2021)	Pregnancy-related hypertension disorder: vaccinated 5.5%, unvaccinated, unvaccinated 4.7%. Placenta abruption: vaccinated 0.3%, unvaccinated 0.3%. Oligohydramnios: vaccinated 2.7%, unvaccinated 3.2%	Caesarean section delivery: vaccinated 19.9%, unvaccinated 3.8%. Postpartum haemorrhage: vaccinated 1.1%, unvaccinated 0.9%.	Respiratory complication: vaccinated 1.5%, unvaccinated 1.8%.
Almutairi et al. (2023)	Placenta abruption: vaccinated 3.5%, unvaccinated 5.3%. Preeclampsia: vaccinated 11.1%, unvaccinated 1.3%. Oligohydramnios: vaccinated 4.8%, unvaccinated 0%.	Caesarean section: vaccinated 43.9%, unvaccinated 51.3%. Postpartum haemorrhage: vaccinated 6.2%, unvaccinated 10.5.	NICU admission: vaccinated 8%, unvaccinated 1.3%. Respiratory distress: vaccinated 4.8%, unvaccinated 0%. 5 min Apgar score < 7: vaccinated 3.5%, unvaccinated 0%.
Condon et al. (2023)	Intrauterine foetal death: vaccinated 0.60%, unvaccinated 0.75%	-	-
Mansour et al. (2023)	Total of 37 spontaneous abortions: 24 received Pfizer-BioNtech (BNT162b2) vaccine 13 received Moderna (mRNA-1273) vaccine	-	-
Fell et al. (2022)	Chorioamnionitis: vaccinated 0.5%, unvaccinated 0.3%	Postpartum haemorrhage: vaccinated 3.0%, unvaccinated 3.4% Caesarean delivery: vaccinated 30.8%, unvaccinated 28.5	NICU admissions: vaccinated 11.0%, unvaccinated 12.8% 5 min Apgar score < 7: vaccinated 1.8%, unvaccinated 2.0%

Note: Please see the full reference list of this article for details on the articles cited: https://doi.org/10.4102/hsag.v29i0.2577 for more information.

NICU, neonatal intensive care unit.

### Ethical considerations

The scoping review was conducted as part of the study, which was approved by the Faculty of Health Science Research Ethics Committee of the University Faculty of Health Science Research Ethics Committee of the University of Pretoria (Ref. no. 709/2022).

## Review findings

The search yielded 871 results, which were then exported to Rayyan. Of the 871 articles, 334 duplicates were removed, and 537 were excluded after screening the titles and abstracts. The full texts of the remaining 27 articles were downloaded and read for in-depth screening. Of the 27 articles, 15 were excluded, and 12 were included in the final review. The PRISMA flow diagram in Figure 1 illustrates the review process.

### Characteristics of the included studies

All 12 studies were conducted in developed countries: eight in Asia and four in the United States. Eight were retrospective, two were cross-sectional and two were prospective. As indicated earlier, the included studies were published between 2020 and 2023. The findings of this review were mapped according to safety during pregnancy, delivery and the neonatal period. The characteristics of the included studies are summarised in Table 1. Table 2 shows the outcomes of the effects of COVID-19 vaccination on pregnant women.

### Coronavirus disease 2019 vaccine safety during pregnancy

Twelve studies reported the safety of the COVID-19 vaccine during pregnancy (Almutairi et al. 2023; Arulappen et al. 2022; Bleicher et al. 2021; Condon et al. 2023; Dick et al. 2022; Fell et al. 2022; Komine-Aizawa et al. 2022; Kugelman et al. 2023; Mansour, Hernandez-Diaz & Wyszynski 2023; Morgan et al. 2023; Rottenstreich et al. 2022; Wainstock et al. 2021), of which nine studies reported that the vaccine is safe and three studies reported a potential safety risk. Pregnancy safety outcomes reported in the studies included postpartum haemorrhage, chorioamnionitis, hypertensive disorder, stillbirth and spontaneous abortions.

A large population-based study conducted in Canada demonstrated that vaccination during pregnancy did not increase the risk of postpartum haemorrhage or chorioamnionitis. This study reported no significant differences in postpartum haemorrhage or chorioamnionitis between the vaccinated and unvaccinated groups (Fell et al. 2022). This finding is consistent with two studies conducted in the United States, which reported that the rate of stillbirth among vaccinated women was not significantly different from that among unvaccinated women (Condon et al. 2023; Morgan et al. 2023)

However, one study (Mansour et al. 2023) reported that 37 spontaneous abortions occurred in the vaccinated group: 13 in the group that received mRNA and 24 in the group that received BNT162b2. This study further revealed that the risk of spontaneous abortion was greater between 6 and 9 weeks of gestation.

Three studies demonstrated that COVID-19 vaccination does not increase the risk of hypertensive disorders (Bleicher et al. 2021; Dick et al. 2022; Komine-Aizawa et al. 2022). However, Almutairi et al. (2023) reported a high rate of hypertensive disorders in the vaccinated group, with 8.3%, compared with 1.3% in the unvaccinated group.

### Coronavirus disease 2019 vaccine safety in terms of delivery outcomes

Seven studies reported delivery outcomes (Almutairi et al. 2023; Arulappen et al. 2022; Dick et al. 2022; Fell et al. 2022; Kugelman et al. 2023; Rottenstreich et al. 2022; Wainstock et al. 2021). The delivery outcomes reported included delivery complications such as preterm delivery, caesarean sections and assisted deliveries, as well as postpartum haemorrhage. Five studies revealed that COVID-19 vaccination does not increase the risk of delivery complications. This was reported by Morgan et al. (2023), who indicated that the rate of preterm delivery before 37 weeks was lower in the vaccinated group (10.3%) than in the unvaccinated group (13.2%). A study conducted in southern Israel revealed no significant differences between vaccinated and unvaccinated groups regarding gestational age at delivery, the number of caesarean sections and vacuum deliveries or postpartum haemorrhage (Wainstock et al. 2021). Furthermore, the study indicated that pregnant women who received two doses of the vaccine were more likely to deliver at a slightly higher gestational age than those who received one dose. In contrast, studies conducted in Jerusalem by Rottenstreich et al. (2022) and in Israel by Wainstock et al. (2021) reported a higher rate of caesarean section and a lower rate of vacuum deliveries among vaccinated women than among unvaccinated women.

### Coronavirus disease 2019 vaccine safety in terms of neonatal outcomes

Eight studies reported neonatal outcomes (Almutairi et al. 2023; Arulappen et al. 2022; Bleicher et al. 2021; Dick et al. 2022; Fell et al. 2022; Morgan et al. 2023; Rottenstreich et al. 2022; Wainstock et al. 2021). The neonatal outcomes reported included neonatal intensive care admission related to preterm birth, low Apgar scores, low birth weights, respiratory complications and small for gestational age. In the Saudi Arabian study, neonatal intensive care unit (NICU) admissions were more common for newborns delivered by vaccinated women, primarily because of low Apgar scores and respiratory complications (Almutairi et al. 2023). However, this was not linked to any specific vaccine type or timing of vaccination and no neonatal deaths were reported. The study further revealed that the average birth weights of newborns from vaccinated and unvaccinated women were similar, but they increased after the second and third vaccine doses. As such, women who received one dose of the vaccine had a lower gestational age at delivery and delivered babies with low birth weight than those who received two doses (Wainstock et al. 2021). A study conducted by Morgan et al. (2023) reported a high rate of preterm birth, with rates of 13% for unvaccinated women and 10.5% for vaccinated women.

### Limitations of the review

This review has several limitations. Although the search strategy was conducted in different databases, certain studies might have been overlooked. Only articles that were published in English were included in this review. This might have affected the interpretation of the results.

**TABLE 3:** Population, concept and context framework.

Criteria	Inclusion	Exclusion
Population	Studies focusing on pregnant women 18 years and older who received the COVID-19 vaccine.	Studies focusing on the general population received the COVID-19 vaccine rather than pregnant women.
Concept	Effect of COVID-19 vaccination.	Studies not answering research the questions.
Context	Studies conducted globally. Published between 2020 and 2023. Published in English. Quantitative and qualitative studies were included.	Studies published in languages other than English. Review articles, commentaries, editorials, and studies published without full text were excluded.

*Source*: Adapted from, Peters, M.D.J., Marnie, C., Tricco, A.C., Pollock, D., Munn, Z., Alexander, L. et al., 2020, ‘Updated methodological guidance for the conduct of scoping reviews’, *JBI Evidence Synthesis* 18(10), 2119–2126. https://doi.org/10.11124/JBIES-20-00167

COVID-19, coronavirus disease 2019.

## Discussion

This study explored the current evidence on the effects of COVID-19 vaccination on pregnant women and neonatal outcomes. The included studies compared the results of pregnancy, delivery and neonatal outcomes between women who had received vaccinations and those who were not vaccinated. Regardless of the type of vaccine received, the results of these studies highlighted the safety of the COVID-19 vaccine in terms of pregnancy and neonatal outcomes. These results are consistent with the expanding body of research that supports COVID-19 vaccination during pregnancy.

The findings of this review revealed no substantial risks associated with receiving the COVID-19 vaccine in terms of pregnancy outcomes concerning stillbirth, intrauterine foetal death, intrauterine growth restriction or any negative effects of gestational age in pregnant women in several studies (Arulappen et al. 2022; Bleicher et al. 2021; Condon et al. 2023). One study revealed that the rates of pregnancy outcomes were similar between vaccinated and unvaccinated pregnant women in terms of hypertensive disorders and anaemia during pregnancy (Bleicher et al. 2021).

In support of the aforementioned, the review further revealed that there was no evidence that COVID-19 vaccination is associated with a high risk of miscarriages or placental abruption (Bleicher et al. 2021; Rottenstreich et al. 2022; Wainstock et al. 2021). These findings from multiple studies provide strong evidence supporting the safety of COVID-19 vaccination during pregnancy with respect to these specific pregnancy outcomes. This findings are consistent with that of Calvert et al. (2022) in a study conducted in Scotland, which reported that there were no evidences that COVID-19 vaccination is associated with high risk of pregnancy complications. Therefore, the findings in this scoping review contribute to a growing body of knowledge that consistently demonstrates that there is no significant risk associated with the COVID-19 vaccine during pregnancy.

The claim that the COVID-19 vaccine has little or no effect on delivery or neonatal outcomes is supported by the lack of significant differences between vaccinated and unvaccinated groups (Almutairi et al. 2023; Kugelman et al. 2023; Rottenstreich et al. 2022; Wainstock et al. 2021). These results support the safety of COVID-19 vaccines in terms of reproductive and neonatal health. These findings are consistent with a previous study that was conducted in China, which reported that COVID-19 vaccination is not associated with increased risk of preterm labour, stillbirth, birth defect as well as neonatal death (Ma et al. 2023). However, Rottenstreich et al. (2022) reported that in Asia, vaccinated women had a greater incidence of Caesarean sections than did unvaccinated women. This inconsistency is concerning and requires additional research and an examination of potential justifications. Furthermore, the differences in C-section rates reported could have been caused by several factors. The increased rate of C-section in the vaccinated group may be influenced by several variables, including differences in maternal characteristics, physician preferences or geographic differences in clinical practice. Therefore, on the basis of these findings, the government should ensure that pregnant women have access to the vaccine because studies have shown that the vaccine is safe during pregnancy and is also beneficial to neonates.

Most studies in the scoping review demonstrated that the COVID-19 vaccine in pregnant women was safe. However, any potential confounding factors that could affect the results should be considered. Given these results, several variables should be considered, including maternal age, comorbidities, socioeconomic position and access to healthcare. Maternal age is an important factor that may impact pregnancy and newborn outcomes. For example, older maternal age has been linked to a greater risk of some pregnancy and delivery problems (Bouzaglou et al. 2020). In addition, maternal comorbidities such as diabetes and hypertension may also have an impact on pregnancy outcomes and may vary between individuals who receive vaccinations and those who do not.

## Conclusion

This scoping review explored the current evidence on the effect of COVID-19 vaccination on pregnant women. On the basis of the evidence presented in this review, COVID-19 vaccination is generally considered safe during pregnancy. Although some studies have reported associations between vaccination and certain conditions, the overall benefits of vaccination outweigh the risks of these conditions. Furthermore, no significant increase in adverse maternal or neonatal outcomes was observed among vaccinated pregnant women compared with unvaccinated women.

The safety and effectiveness of COVID-19 vaccines during pregnancy should be continuously monitored through ongoing research. Therefore, pregnant women should consult with their healthcare providers to make informed decisions regarding COVID-19 vaccination. Governments and health organisations should prioritise efforts to better inform women of reproductive age about COVID-19 vaccination during pregnancy.
